# Brain–immune–gut benefits with early life supplementation of milk fat globule membrane

**DOI:** 10.1002/jgh3.12775

**Published:** 2022-06-01

**Authors:** Hamid Jan Jan Mohamed, Eric Kim Hor Lee, Kent Chee Keen Woo, Rajini Sarvananthan, Yeong Yeh Lee, Zabidi Azhar Mohd Hussin

**Affiliations:** ^1^ Nutrition and Dietetics Programme, School of Health Sciences Universiti Sains Malaysia Kelantan Malaysia; ^2^ Pantai Hospital Kuala Lumpur Malaysia; ^3^ Allergy and Immunology Clinic Gleneagles Hospital Kuala Lumpur Malaysia; ^4^ ParkCity Medical Centre Kuala Lumpur Malaysia; ^5^ School of Medical Sciences Hospital Universiti Sains Malaysia Kelantan Malaysia; ^6^ School of Medicine International Medical University Kuala Lumpur Malaysia

**Keywords:** brain–gut axis, human milk, immunity, infant formula

## Abstract

The milk fat globule membrane (MFGM) has been recognized as a milk component for more than 60 years, but its exact benefits remain unknown. Research on human MFGM has revealed that the membrane holds a host of bioactive components with potential benefits for the brain–immune–gut (BiG) axis in early life. Gangliosides and sphingomyelin, components within the MFGM, have been included in infant formulas for many years. Recent advancements in dairy milk processing have allowed the successful separation of MFGM from bovine milk, enabling it to be used for supplementing infant formulas. Evidence indicates the potential benefits of MFGM in early life supplementation, including better cognitive development, reduction of infection risks, and modulation of the gut microbiome. However, larger and more robust randomized trials are needed, in addition to long‐term outcome data beyond the infancy period.

## Graphical abstract

The milk fat globule membrane (MFGM) has been recognized as a milk component for more than 60 years, but its exact benefits remain unknown. Research on human MFGM has revealed that the membrane holds a host of bioactive components with potential benefits for the brain–immune–gut axis in early life. Evidence indicates the potential benefits of MFGM in early life supplementation, including better cognitive development, reduction of infection risks, and modulation of the gut microbiome.

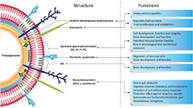

## Introduction

Continuing research on the composition human milk over the decades has increased the understanding of its nutrients in maintaining healthy infant growth and development. This has, in turn, helped in the advancement of infant formula development.

Though the milk fat globule membrane (MFGM) inherently makes up only a small part of all cow milk‐based products, components of MFGM, namely sphingomyelin (SM) and gangliosides, have been added to infant formulas for some years now, gaining familiarity among healthcare professionals (HCPs). However, the supplementation of MFGM as a whole in infant formulas is relatively new, primarily contributed by identifying the beneficial effects of the individual MFGM components. Recent advances in dairy technology have allowed the separation of MFGM from the fat globule, enabling the production of bovine MFGM to be available for use in infant formulas.^1^, ^2^

This article aims to provide viewpoints on the role of MFGM supplementation in early life on the brain–immune–gut (BiG) axis, by understanding its structure and function and the benefits of adding MFGM in infant formulas, as well as identifying evidence gaps in the clinical benefits of MFGM supplementation and desired future research.

## Methods

The content for this review was formulated during a series of virtual workshops conducted in late 2020 with experts (listed as authors), which included a general pediatrician, a pediatric nutritionist, an allergist and immunologist, a pediatric gastroenterologist, a pediatric neurologist, and a developmental pediatrician. The PubMed database was used to collect articles that were in the English language and filtered according to original articles of clinical trials, meta‐analyses, randomized clinical trials, reviews, and systematic reviews. The search criteria did not set limits to time and included all relevant articles from the beginning to the time of search (March 2022). The following search terms were used alone: “milk fat globule membrane,” “human milk fat globule membrane,” and “bovine milk fat globule membrane.” The term “milk fat globule membrane” was also used in combination with the following terms: “brain,” “cognition,” “immune,” “gut,” “early life,” “infant,” “formula,” “dairy,” “bovine,” “structure,” “function,” “effects,” and “benefits.” A total of 755 articles were retrieved. Duplicate articles were removed and only articles pertaining to full‐term infants and supplementation with MFGM alone were reviewed.

### MFGM structure and function

**Figure 1:**
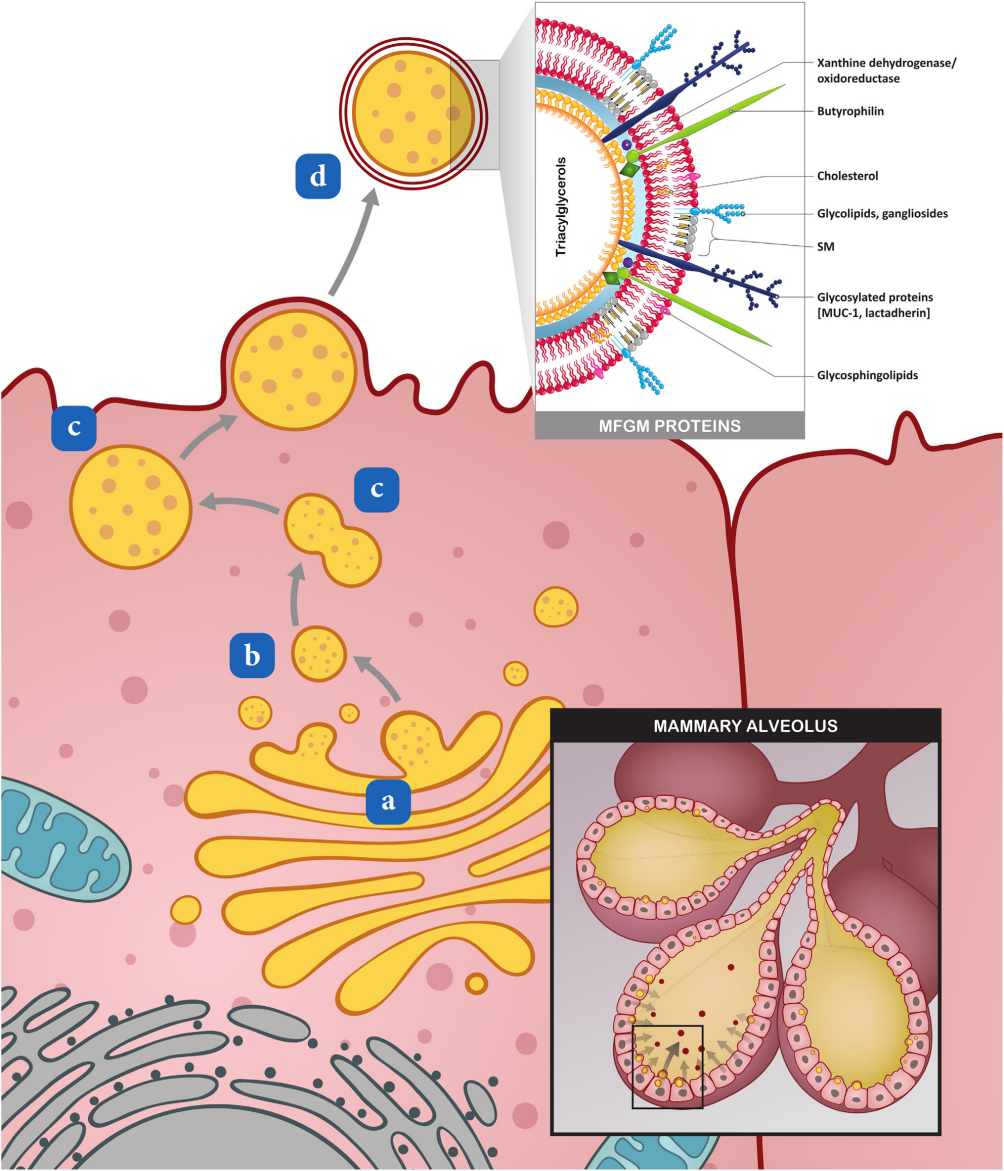
Formation of the MFG with its tri‐layer membrane (the MFGM). (a) Small lipid droplets are formed within the endothelial reticulum (ER) of the mammary cells. (b) By the process of budding, they break off from the ER and enter the mammary cell cytoplasm. (c) These single membrane lipid droplets coalesce to form larger lipid droplets. (d) Excretion of these larger single membrane lipid droplets occurs at the apical cell surface of the mammary cells. The double‐layered plasma membrane is pinched off as the droplet exit, resulting in a tri‐layer envelope around the newly formed MFG. MFG, milk fat globule, MUC‐1, mucin‐1; SM, sphingomyelin.

**Figure 2:**
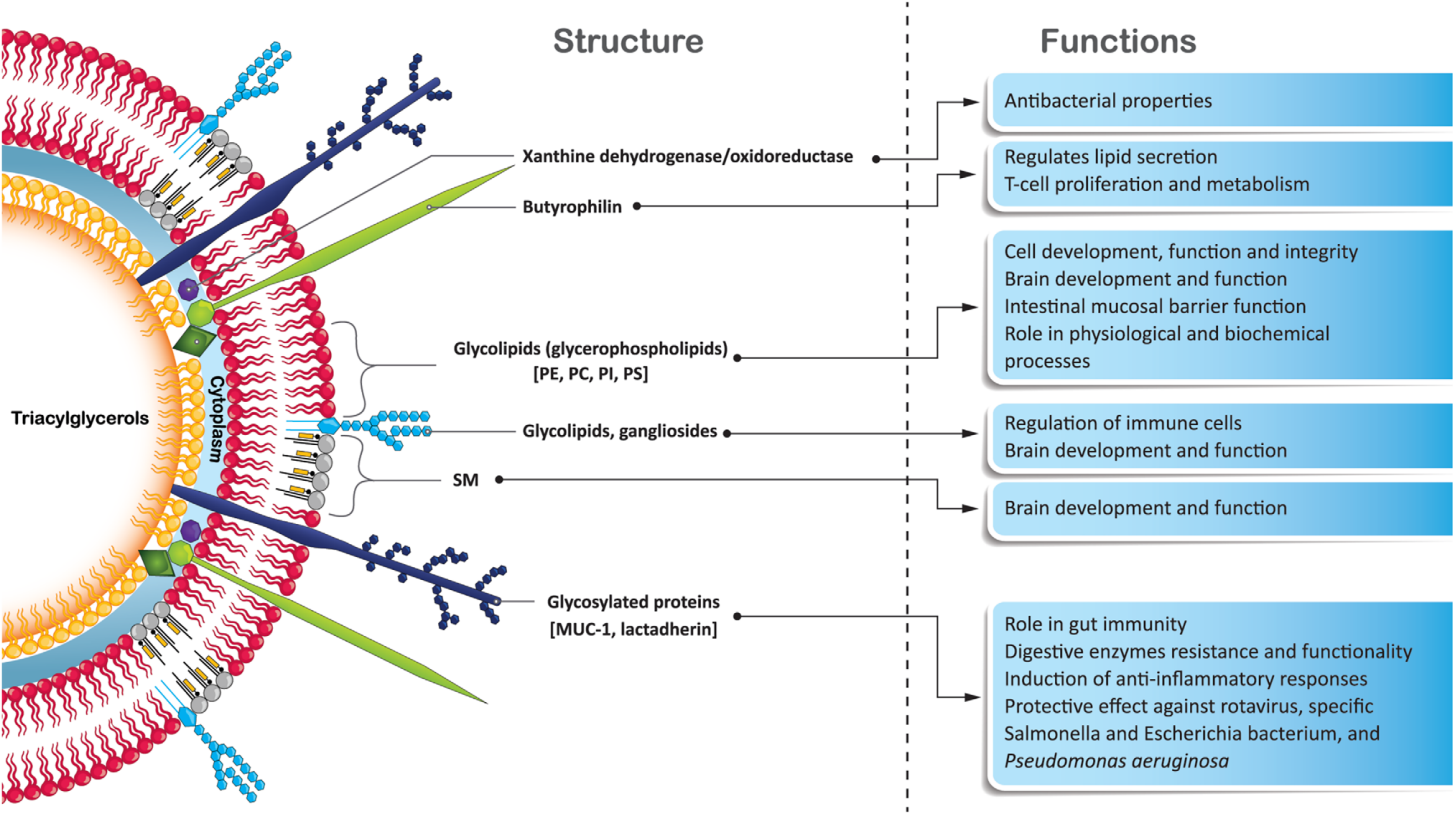
Functions of MFGM proteins and lipids. A simplified illustration depicting some of the MFGM components mentioned in this article and their associated functions.^4^, ^5^, ^6^, ^7^, ^8^, ^9^ MUC‐1, mucin‐1; PC, phosphatidylcholine; PE, phosphatidylethanolamine; PI, phosphatidylinositol; PS: phosphatidylserine; SM, sphingomyelin.

Human milk fat is produced by the formation of lipid droplets within the mammary cells, which are released from the mammary cells in the form of milk fat globules (MFGs) (Fig. 1).^3^ The core of the globule consists of triacylglycerols, while its membrane (i.e., MFGM) consists of three layers. The membrane functions to stabilize the globule as an emulsion, and is a complex structure containing a variety of different components, of which many, but not all, have been characterized.^3^ The two primary components of the MFGM are proteins and lipids. The lipids make up 40% of the MFGM, and proteins 60%.^2^, ^4^ The functions of specific MFGM components are briefly depicted in Figure 2.

The lipid component of MFGM consists of phospholipids, glycolipids (polar lipids), gangliosides, cholesterol, and other lipids.^10^, ^11^ Phospholipids are the major lipid component of MFGM (~30% of the total lipid weight of MFGM). It only makes up 0.5–1% of the total fat in milk, yet it contains 15–20% of the total long‐chain polyunsaturated fatty acids.^10^, ^12^ The MFGM contains five major glycolipids: phosphatidylcholine (PC), phosphatidylethanolamine (PE), phosphatidylinositol (PI), phosphatidylserine (PS), and sphingomyelin (SM). SM, PC, and PE combined form up to 85% of the polar lipids. High‐performance liquid chromatography has elucidated that PC and SM are made up of different subclasses.^13^, ^14^ Glycolipids play an essential role in the development of the central nervous system and within the gut and immune systems.^15^, ^16^, ^17^ The functions of each glycolipid component are described in Table 1.

**Table 1:** Brief description of the components and functions of the MFGM glycolipids

Glycolipid components	Composition in human milk glycolipids	Function
SM^6^, ^18^, ^19^	36–38% of glycolipids in human milk	Supports myelination of the neuron; Important for cell–cell and cell–matrix interactions, cell adhesion, modulation of membrane receptors and signal transduction.
PC^6^, ^7^	25–28% of glycolipids in human milk	A major structural component of cell membranes; A significant source of choline; It is involved in the synthesis of SM and acetylcholine; Plays a role in intestinal mucosal barrier function.
PE^6^, ^7^, ^8^	20–29% of glycolipids in human milk	Required for the synthesis of PS; Essential in cell development, function, and integrity; A necessary precursor for PC.
PS^6^, ^7^, ^8^	6–8% of glycolipids in human milk	Synthesized from PC and PE; Contributes to the integrity of the membrane; Found abundantly in the gray matter of the brain together with DHA; Plays a role in neuronal survival and differentiation and neurotransmitter release.
PI^6^, ^7^, ^20^	~5% of glycolipids in human milk	Major inositol‐containing phospholipid in cells; Primary function is at the cell membrane level; Responsible for various physiological and biochemical processes (e.g., the response of cells to hormones and neurotransmitters to produce fast physiological responses or stimulate cell proliferation); May have a role in synaptic transmission processes.

DHA, docosahexaenoic acid; MFGM, milk fat globule membrane; PC, phosphatidylcholine; PE, phosphatidylethanolamine; PI, phosphatidylinositol; PS, phosphatidylserine; SM, sphingomyelin.

MFGM carries between 1 and 4% of the total protein fraction in milk, although the levels vary along the lactation timeline. To date, 191 different bioactive proteins have been identified in human MFGM, which contribute to growth functions or are involved in various lipid metabolic processes or immunity.^21^ The MFGM protein includes glycosylated proteins, which may play a role in gut immunity, determine resistance to digestive enzymes, and functionality in the gut. It also includes non‐glycosylated proteins such as xanthine oxidoreductase, an enzyme found in large amounts within the MFGM and is shown to exert an antimicrobial effect due to its role in the production of reactive oxygen species.^5^, ^10^

### Are MFGMs sourced from human milk, bovine milk, and dairy products different?

On one hand, MFGM in human and bovine milk probably share many similarities with some distinct features (Table 2). On the other hand, the structure and composition of MFGM can vary widely between dairy products and human or bovine milk. The size of the milk fat globule, regardless of its source, is influenced by various factors such as (i) genetics, (ii) diet (the more the fat, the larger the globule), (iii) parity, and (iv) stage of lactation.^10^, ^13^, ^22^ While most milk (either bovine or human) contains medium‐sized fat globules, milk with larger globules have a smaller surface area, resulting in less available MFGM; the converse is true for smaller globules.^1^, ^22^ The phospholipids level and their components in bovine and human milk are mostly comparable despite scarce and sometimes conflicting data, while the protein composition is similar in both.^24^, ^25^ Based on this, bovine‐derived MFGM may be considered appropriate if added to infant formulas.

**Table 2:** Differences between human and bovine milk MFGM

Parameter	Human milk	Bovine milk
Size of MFG^14^, ^22^	0.35–13 μm	0.1–15 μm (2–5 μm in common livestock species)
Phospholipids within MFGM^10^, ^22^	Predominantly SM; The SM is of a higher quality compared to bovine milk; PC and PE are the second most predominant; PS and PI are minor components.	The proportion and composition vary depending on animal species and size of the MFG; Smaller globules have a higher proportion of the five different classes; Larger globules have more PC and SM (e.g., in whole milk).
LC‐PUFAs^10^, ^22^	High content within MFGM; 15–20% of total LC‐PUFAs.	High content within MFGM, particularly in cow and sheep milk.
Proteins^1^, ^23^	191 bioactive protein compounds identified.	120 protein components identified; These include enzymes, immunoglobulins and skim milk constituents that may exert biological functions.

LC‐PUFA, long‐chain polyunsaturated fatty acid; MFG, milk fat globule; MFGM, milk fat globule membrane; PC, phosphatidylcholine; PE, phosphatidylethanolamine; PI, phosphatidylinositol; PS, phosphatidylserine; SM, sphingomyelin.

When milk is processed during the production of dairy products, MFGM is disrupted, and aqueous separation of the fat globule occurs.^18^, ^26^ Additionally, processing of milk, with processes such as centrifugation, heating, cooling, and storage, leads to loss of the phospholipid fraction in MFGM.^26^ There is also absorption of the casein fraction into the disrupted MFGM, which may affect milk digestion. Because of this, buttermilk and butter serum in dairy are rich sources of MFGM components^18^ and hold better potential if incorporated into infant formulas.^11^

### What are the factors to consider in commercial MFGM‐supplemented infant formula?

The rationale in adding MFGM (as a whole) into infant formula is to harness the benefits of all its nutrients.^23^ However, commercially available MFGM‐enriched dairy ingredients will differ in the quantity of phospholipids and proteins and in the composition of the five key glycolipids depending on their source.^23^ Most commercially available infant formulas use non‐fat milk as its base and vegetable oils to provide its fat content, often resulting in low MFGM levels.^27^ When the fat globule in human milk is compared with that in processed lipid droplets in commercial infant formulas, there are several notable differences.^28^

First, the lipid droplets in commercial infant formulas are much smaller than in human milk (0.3–0.8 *vs* 0.4–13 μm). Second, the human milk fat globule is covered by the three‐layered MFGM. In contrast, lipid droplets in infant formulas are covered by milk proteins, resulting in the lack of rich milk SM, other glycolipids, cholesterol, and glycoproteins commonly found in nature. Third, protein aggregates in infant formula are formed between the lipid droplets and proteins from heat‐induced denaturation during milk processing. This is further influenced by the blending of fats from various sources. Fourth, the levels of MFGM in an infant formula will vary depending on the manufacturers.^28^

The bioavailability of MFGM from infant formulas is indirectly obtained from preclinical and clinical studies. Infants supplemented with bovine MFGM have shown significantly different plasma lipidome profile (particularly SM, PC, and ceramides) at 6 months of age, as well as in the erythrocyte membrane lipidome (particularly SM, PE, and PC).^29^ Most preclinical studies reveal that complex milk lipids could enhance brain and neuronal development, leading to better cognitive outcomes.^19^, ^30^, ^31^ In terms of immunity development, anti‐rotaviral activity has been demonstrated from the lipid components of MFGM^32^ and bovine MFGM‐linked mucin protein.^33^ MFGM glycoproteins have shown better resistance to gastrointestinal proteases *versus* non‐glycosylated proteins, which may be due to the presence of lipids within the MFGM structure.^34^

### Are there brain–immune–gut (BiG) benefits with MFGM supplementation?

Multiple reports have associated the positive effects of different MFGM bioactive components (in human and bovine milk) with cognitive and immunity development of infants, without impairing their growth.^35^, ^36^, ^37^, ^38^, ^39^, ^40^, ^41^

A randomized controlled trial (RCT) involving 160 healthy term infants <2 months old compared standard formula (SF) to a formula containing bovine MFGM (Lactoprodan MFGM‐10, Arla Foods Ingredients) (experimental formula, EF). The infants were fed formula exclusively up to age 6 months, and followed up until 12 months. Cognitive scores (*Bayley Scales of Infant and Toddler Development*, 3rd edition [Bayley‐III] test) at 12 months showed that EF scored 4.0 points higher *versus* SF (95% CI 1.1, 7.0; *P* = 0.008), and a score similar to that of exclusively breastfed (BF) infants (<1 point difference; *P* = 0.35).^38^ In the same cohort of infants, EF also showed positive outcomes with immunity; cumulative incidence of acute otitis media at 6 months was lower with EF than with SF (1 *vs* 9%; *P* = 0.034), and also, the use of antipyretics through 6 months was lower with EF than with SF (25 *vs* 43%; *P* = 0.021). A limitation of this study was the subjective reporting of symptoms by parents, although acute otitis media was verified from medical records. The low incidence of infections in the study population may also be of significance; the outcomes of the intervention in a population with a higher infectious morbidity may be of a different magnitude.^36^ Furthermore, the microbiome in the oral cavity was studied in the same cohort, and the pathogen *Moraxella catarrhalis* was found to be lower in the EF group, which may have contributed to the lower incidence of acute otitis media.^42^ However, follow‐up when the children were 6–6.5 years old revealed no effects on neurodevelopment, growth, or plasma cholesterol status between the EF and SF groups.^43^

Another RCT compared SF and SF + MFGM (Lactoprodan MFGM‐10) + lactoferrin formula (EF) in 451 healthy term infants aged 10–24 days. The infants were exclusively fed the formula up to day 120, after which parents could introduce complementary feeding. Stage 1 formula was switched to the corresponding stage 2 formula from day 180 through day 365. Cognitive scores (Bayley‐III test) at day 365 showed significantly higher mean scores in cognitive, language, and motor domains for EF than SF (+8.7, +12.3, and +12.6, respectively; *P* < 0.001). EF also showed improvement in global developmental and attention scores *versus* SF. Significantly lower incidences of upper respiratory tract infection (URTI; 68% *vs* 78%; *P* = 0.02), cough (40 *vs* 30%; *P* = 0.02), and diarrhea (68 *vs* 54%; *P* = 0.003) were observed with EF than SF, whereas no differences were observed in other adverse events such as eczema, constipation, fussiness, and amount of gas. A potential limitation to this study was that the individual effects of MFGM and lactoferrin on the outcomes in the EF group could not be distinguished with certainty.^35^

An RCT randomized 212 healthy term infants aged <14 days to receive either a bovine MFGM‐enriched formula (EF) or a control formula (Stage 1 provided at 0–6 months, and then switched to Stage 2 at 6–12 months). The EF group scored higher in composite social emotional (+3.5) and general adaptive behavior (+5.62) at 12 months of age *versus* the control group (95% CI 0.03–6.79 and 1.78–9.38; *P* = 0.048 and 0.004, respectively). Short‐term memory score was significantly higher in the EF group at 12 months of age (95% CI 1.40–12.33; *P* = 0.002), and serum gangliosides level was significantly higher in the EF group at 4 months of age (95% CI 0.64–13.02; *P* = 0.025).^39^

The COGNIS study was an RCT analyzing 70 children who were fed SF or an experimental infant formula (EF) containing MFGM components, synbiotics, long‐chain polyunsaturated fatty acids, gangliosides, sialic acid, and nucleotides during the first 18 months of life and 33 BF children (reference group). The EF‐fed children at 2.5 years presented fewer pathological affective problems than SF‐fed children. Rates of externalizing problems were increased in SF infants *versus* EF and BF infants.^40^

An RCT studied the impact of SF or SF supplemented with probiotic or MFGM (Lactoprodan MFGM‐10) (EF) in 600 healthy term infants aged 21 ± 7 days old, followed up until 4 months of age. Episodes of fever >38°C were fewer with EF *versus* SF (11 *vs* 21, *P* = 0.242), as was number of days with fever (18 *vs* 31, *P* = 0.230), although both associations were not significant. A limitation of this study in analyzing the impact of MFGM supplementation in infant immunity was that no otitis media assessment was performed.^44^

An RCT involving 550 healthy term infants aged 6–11 months compared 6 months of complementary food + MFGM protein fraction (Lactoprodan MFGM‐10) or skim milk proteins (control). The prevalence of diarrhea was lower in infants fed complementary foods supplemented with MFGM than in those without supplementation (3.84 *vs* 4.37%; *P* < 0.05). MFGM supplementation also reduced bloody diarrhea episodes (odds ratio [OR] 0.54; 95% CI 0.31–0.93; *P* = 0.025).^37^

An RCT randomized formula‐fed infants to either a formula with probiotic *Lactobacillus paracasei* ssp. paracasei strain F19 (*N* = 195) or MFGM (*N* = 192), or standard formula (SF) (*N* = 194) from age 21 ± 7 days until 4 months. A BF group served as reference (*N* = 208). The MFGM group had lower IL‐2 and IL‐17A concentrations compared with the SF group (*P* < 0.05). Cytokine concentrations were comparable between the MFGM and BF groups. The study concluded that the cytokine profile of the MFGM group approached that of BF infants, which might explain why the infectious outcomes for the MFGM group in this cohort were closer to those of BF infants, compared to SFs. The study limitations included the regional differences in immunization programs and the lack of a blood sampling at baseline for comparison of cytokine responses before and after the intervention period.^41^

An RCT characterized the fecal microbiome of 90 infants who were fed either a bovine MFGM‐supplemented experimental formula (Lacprodan MFGM‐10) (EF), an SF, or BF. All infants showed a high prevalence of *Bifidobacterium*, *Streptococcus*, *Veillonella*, *Enterococcus*, and *Bacteroides* from 2 to 6 months of age. Fecal *Bifidobacteria* and other microbes were significantly different between BF and formula‐fed infants, but the difference between EF and SF was small. By 12 months of age, the fecal microbiome became more homogenous and indistinguishable between BF and formula‐fed infants. In order to evaluate the impact of diet on intestinal microbial fermentation capability, the fecal metabolites were examined as well. A significant difference between the BF and formula‐fed infants was observed from 2 months of age, which continued at 4 and 6 months when no or only small amounts of complementary food were introduced. Similar to the microbial profile, BF infants had a more heterogeneous fecal metabolome profile than formula‐fed infants. The difference in fecal metabolome was less significant in infants who consumed complementary food and was indistinguishable by 12 months of age.^45^

Allergy benefits associated with MFGM supplementation are unclear, and though three studies^35^, ^36^, ^44^ showed no significant reports of eczema with the use of MFGM supplemented formulas, one study^46^ revealed a higher incidence of eczema in the protein‐ *versus* lipid‐rich MFGM formula.

Table 3 provides an overview of the clinical benefits of MFGM supplementation associated with the BiG axis of developing term infants. Bovine MFGM supplementation has been shown to narrow the gap in immunologic and cognitive development between BF and formula‐fed infants. Therefore, it would not be amiss to consider MFGM supplementation in children in addition to more “traditional” nutrients such as docosahexaenoic acid (DHA) and arachidonic acid (ARA), to ensure the best possible conditions for wholesome childhood development. In healthy term infants, MFGM supplementation, in addition to protein, iron, and ARA, was well tolerated, associated with adequate growth throughout the first year of life, and supported normal iron status at 1 year of age.^47^

**Table 3:** Summary of benefits of MFGM supplementation on the brain–immune–gut (BiG) axis and the microbiome in infants

Brain	Immunity	Gut	Microbiome
Improved cognitive scores (cognitive, language and motor domains)^35^, ^38^ Improved developmental and attention scores^35^ Narrowed the gap in cognitive development between breastfed and formula‐fed infants^38^ Improved social, emotional, and general adaptive behavior scores^39^ Improved short‐term memory score^39^ Fewer pathological affective problems and externalizing problems^40^	Reduced otitis media risk and decreased antipyretic use^36^ Fewer incidences of URTI, cough, and diarrhea^35^ Lower IL‐2 and IL‐17A concentrations^41^ Cytokine profile approaches that of BF infants^41^	Fewer diarrheal episodes^35^, ^37^ Reduced bloody diarrhea episodes^37^	Shaped gut microbial activity and function^10^ Lower prevalence of *Moraxella catarrhalis*, which is associated with otitis media^42^

BF, breast‐fed; IL, interleukin; URTI, upper respiratory tract infections.

### What are the evidence gaps and suggestions for future clinical research?

Available efficacy and safety data show a tendency of MFGM supplementation to improve cognitive scores and immunity (Table 3). However, in most studies the sample sizes were small and heterogenous in their methodology, which pose a challenge.^48^ To address cognitive benefits, cross‐over double‐blinded studies may be used to assess social and emotional scores. Though there are ongoing studies on the clinical outcomes of MFGM, there is a lack of long‐term data to confirm a sustained cognitive benefit of MFGM in children beyond 1.5 years of age. Additionally, there is a lack of replicated studies to further strengthen the currently available evidence. MFGM levels and composition in different populations may also differ. For example, a study on ganglioside levels in human milk of Malaysian mothers has shown a higher concentration than those previously reported in other populations.^49^ The results also indicate a relationship between ganglioside levels and fat content in the milk: higher fat content results in higher ganglioside levels. Although there can be variations in ganglioside levels in human milk attributable to factors such as sampling time, collection method, demographics, diet, and stage of lactation, the level of gangliosides found in infant formulas is generally lower than in human milk.^49^, ^50^ Therefore, considering that human milk is the gold standard against which infant formulas are formulated, the authors concluded that these findings could support the need for increasing the lipid components (e.g., gangliosides, phospholipids) in infant formulas to further approximate those found in human milk.^49^ In addition, it may be advantageous to determine whether there is a “window of opportunity” in the infancy period when MFGM supplementation would be most impactful. This may help doctors guide parents on when best to use MFGM‐supplemented infant formulas in an ecosystem overwhelmed with formulas supplemented with a host of bioactives. A new area that would be of potential interest to clinicians is the effect of MFGM on the gut microbiome. Studies on the metabolome would be helpful, as byproducts of microbiome metabolism are closely linked with gut immunity.^45^ Besides, expanding MFGM research to determine its clinical benefits in specific cohorts of infants such as those with allergies or at increased risk of infection may be of interest to clinicians.

## Conclusion

Human milk is the best single source of MFGM for infants; however, when this is not an option, a formula that is closer in makeup to human milk may be considered. Available commercial formulas differ in the level and composition of MFGM, and thus may affect bioavailability and clinical benefits. Current evidence indicates that MFGM has the potential to benefit the BiG axis in infants through better cognitive development, reduced infection risks (acute otitis media, respiratory tract infections, and diarrhea), and probably shaping the gut microbiome activity and function.
